# Biochemical and Molecular Analysis of *Staphylococcus aureus* Clinical Isolates from Hospitalized Patients

**DOI:** 10.1155/2016/9041636

**Published:** 2016-04-24

**Authors:** Amit Karmakar, Parimal Dua, Chandradipa Ghosh

**Affiliations:** Microbiology Laboratory, Department of Human Physiology with Community Health, Vidyasagar University, Paschim Medinipur, West Bengal 721102, India

## Abstract

*Staphylococcus aureus* is opportunistic human as well as animal pathogen that causes a variety of diseases. A total of 100* Staphylococcus aureus* isolates were obtained from clinical samples derived from hospitalized patients. The presumptive* Staphylococcus aureus* clinical isolates were identified phenotypically by different biochemical tests. Molecular identification was done by PCR using species specific 16S rRNA primer pairs and finally 100 isolates were found to be positive as* Staphylococcus aureus*. Screened isolates were further analyzed by several microbiological diagnostics tests including gelatin hydrolysis, protease, and lipase tests. It was found that 78%, 81%, and 51% isolates were positive for gelatin hydrolysis, protease, and lipase activities, respectively. Antibiogram analysis of isolated* Staphylococcus aureus* strains with respect to different antimicrobial agents revealed resistance pattern ranging from 57 to 96%. Our study also shows 70% strains to be MRSA, 54.3% as VRSA, and 54.3% as both MRSA and VRSA. All the identified isolates were subjected to detection of* mec*A,* nuc*, and* hlb* genes and 70%, 84%, and 40% were found to harbour* mec*A,* nuc*, and* hlb* genes, respectively. The current investigation is highly important and informative for the high level multidrug resistant* Staphylococcus aureus* infections inclusive also of methicillin and vancomycin.

## 1. Introduction

*Staphylococcus aureus* is one of the most harmful species of staphylococci encountered. It is the leading cause of bacteremia, pneumonia, myocarditis, acute endocarditis, pericarditis, osteomyelitis, encephalitis, meningitis, chorioamnionitis, mastitis, and scalded skin syndrome [1]. Human morbidity and mortality in hospital settings are largely caused by staphylococcal bacteremia [2]. The pathogenic capacity of* Staphylococcus aureus* is clearly dependent on its production of exoproteins and toxins. The species is identified on the basis of a variety of conventional physiological or biochemical characters. The key characters for* Staphylococcus aureus* are colony pigment, free coagulase, clumping factor, protein A, heat-stable nuclease, lipase, and acid production from mannitol [1]. In addition,* Staphylococcus aureus* can be identified by PCR methods. 16S rRNA gene is reported to be the most useful and extensively investigated taxonomic marker molecules [3].

Due to excessive and uncontrolled use of antibiotics the organism becomes multidrug resistant leaving few therapeutic options for the treatment against it [4]. The emergence of methicillin-resistant* Staphylococcus aureus* (MRSA) in hospital-acquired (HA) infections highlights this species as a potential pathogen that is able to cope with the antimicrobial agent [5]. The* mec*A gene [6, 7] synthesizes penicillin binding protein (PBP2a) and it is the cause of methicillin resistance in MRSA. This gene resides on the staphylococcal cassette chromosome (SCC) [8]. Staphylococcal cassette chromosome is a large genetic mobile element which varies in size and genetic composition among the strains of MRSA [9]. Colonized hospital personnel and infected patients are the main source of MRSA [10]. Knowledge of selection of the antibiotics for treatment is important as antibiotic responsiveness pattern of MRSA may vary from region to region [11]. To treat staphylococcal infections, various classes of antibiotics including beta-lactams, glycopeptides, lipopeptide, oxazolidones, aminoglycosides, macrolides, and fluoroquinolones were used [12–15]. Resistance of staphylococci occurs with a variety of antimicrobial agents as well as vancomycin. Therefore, vancomycin resistance is another problem for the treatment of infections caused by this bacterium [16].

The present work was planned to identify and characterize* Staphylococcus aureus* isolates collected from postoperative wound swab of male and female patients hospitalized in the various departments of Midnapore Medical College and Hospital, Midnapore, West Bengal, India. The objectives of the present study were to find out the rate of MRSA and vancomycin resistant* Staphylococcus aureus* (VRSA) in hospital-acquired infections and to determine the antibiotic responsiveness pattern.

## 2. Materials and Methods

### 2.1. Bacterial Isolates and Culture Methods

From January 2013 to October 2014, a total of 165 nonduplicate bacterial isolates were collected from the Male Surgical Wards, Orthopedic Ward, and Burn and Wound Section of Midnapore Medical College and Hospital, Midnapore, West Bengal, India. Only strains obtained from a pure culture were included. Only the first strain from each patient was included. All the strains were collected aseptically, transferred into mannitol salt agar (MSA) media, HiMedia (Mumbai), and incubated overnight at 37°C [17].

### 2.2. Isolation and Phenotypic Identification of* Staphylococcus aureus*

Each isolated bacterial sample from MSA media was initially evaluated on 5% sheep blood agar and incubated overnight at 37°C. Each culture underwent Gram staining and was tested for production of catalase, free coagulase, yellow pigment, and thermonuclease (TNase), according to Lancette and Tatini [18].

### 2.3. Molecular Identification through PCR Amplification of 16S rRNA Gene

The bacterial isolates which were found to be* Staphylococcus aureus* by specific phenotypic features were further tested by PCR for confirmation using specific primer pairs of 16S rRNA (Table 1). These primers amplify 228 bp region of 16S rRNA gene fragment of* Staphylococcus aureus*, which is highly conserved at species level. PCR programming was begun with an initial denaturation step at 94°C for 2 min followed by 35 cycles of 94°C for 30 sec, 50°C for 30 sec, and 72°C for 45 sec, ending with a final extension step at 72°C for 4 min.* S. aureus* ATCC 25923 was used as positive control in this experiment.

### 2.4. Biochemical Characterization of* Staphylococcus aureus* Strains

The bacterial strains that were confirmed as* Staphylococcus aureus* by species specific 16S rRNA screening were further analyzed by several microbiological diagnostics tests including mannitol fermentation and growth on high salt concentration, gelatin hydrolysis, urea hydrolysis, protease activity on milk agar medium, lipase production on egg yolk agar medium (HiMedia, Mumbai), and hydrolysis of esculin by standard methods [23–26]. Hemolytic activity was determined on sheep blood agar (15 mL of 5% sheep blood in Trypticase soy agar overlaid on 10 mL of blood agar base) according to Rodgers et al. [27].

### 2.5. Antibiotic Susceptibility Pattern of Isolated* Staphylococcus aureus* to Some Antimicrobial Agents and Detection of VRSA

The susceptibility of the* Staphylococcus aureus* isolates to different antimicrobial agents was done by disk diffusion method using commercial disks obtained from HiMedia, Mumbai [28]. The antimicrobial disks were as follows: ampicillin (30 *μ*g), streptomycin (10 *μ*g), kanamycin (30 *μ*g), erythromycin (10 *μ*g), chloramphenicol (30 *μ*g), cefoxitin (30 *μ*g), nalidixic acid (30 *μ*g), and novobiocin (30 *μ*g). Clinical strains were categorized as susceptible and resistant according to evaluation criteria developed by the Clinical and Laboratory Standards Institute (CLSI) guidelines [29]. To identify VSSA and VRSA, MIC of vancomycin was determined by the microbroth dilution method using the Mueller-Hinton broth according to the Clinical Laboratory Standards Institute guidelines [29].* Staphylococcus aureus* ATCC 29213 strains were used as vancomycin-susceptible controls and* Enterococcus faecalis* ATCC 51299 as vancomycin resistant control.

### 2.6. Detection of* mec*A,* nuc*, and* hlb* Genes

*Staphylococcus aureus* isolates were subjected to the detection of* mec*A,* nuc*, and* hlb* genes by PCR using specific primer pairs (Table 1). The amplification was performed in a Thermal Cycler (Eppendorf, Germany) beginning with an initial denaturation step at 95°C for 2 min followed by 35 cycles of 94°C for 1 min, 55°C for 45 sec, 50° for 50 sec, and 52° for 40 sec for* mec*A,* nuc*, and* hlb* gene, respectively, and a common extension for 72°C for 2 min, ending with a final extension step at 72°C for 4 min. For rapid DNA extraction from* Staphylococcus aureus* one to five bacterial colonies were suspended in 50 *μ*L of sterile Milli Q water (Millipore-Synergy®, India) and heated at 99°C for 10 min in a 500 *μ*L Eppendorf tube. After centrifuge at 14000 r.p.m. for 10 min in a mini centrifuge (Eppendorf, Germany), 2 *μ*L of the supernatant was used as template.

## 3. Results

In the present study 165 nonduplicate samples were collected from hospitalized patients. Among 165 samples, 100 strains (60.60%) were isolated from a selective MSA media and then these isolates were identified for* Staphylococcus aureus* by different biochemical tests (Table 2). Gram staining, catalase, coagulase, and thermonuclease were important phenotypic identifying markers of* Staphylococcus aureus*. In this study we found that 100%, 92%, and 84% isolates were positive for catalase, coagulase, and heat-stable nuclease, respectively (Table 2). All* Staphylococcus aureus* isolates (100) in this study were confirmed by PCR using the species specific primer pair (Table 1). The enzyme gelatinase was secreted by* Staphylococcus aureus* liquefy gelatin protein. Present study also showed that 78% of* Staphylococcus aureus* isolates were able to produce gelatinase. In Table 3, 81%, 51%, and 48% of isolated strains produced protease, lipase, and nonwhite pigmented colonies, respectively. Present study showed that 40% of* Staphylococcus aureus* isolates were able to produce clearing zone surrounding their growth on blood agar media (Table 4) demonstrating that they can produce hemolysin. Rates of resistance pattern of* Staphylococcus aureus* ranged from 57 to 96%. Resistance of* Staphylococcus aureus* isolates to ampicillin (96%), cefoxitin (70%), kanamycin (74%), erythromycin (81%), streptomycin (75%), nalidixic acid (89%), novobiocin (75%), and chloramphenicol (57%) was found (Table 5). All* Staphylococcus aureus* strains isolated in this study were found to be multidrug resistant. In the present study we have found that the prevalence rate of MRSA is 70%. Our study also found that 54.3% of the isolated* Staphylococcus aureus* were vancomycin resistant (Table 6) and 54.3% of the methicillin-resistant* Staphylococcus aureus* were resistant to vancomycin. According to the CLSI guideline [29] strains having a vancomycin MIC of 4–8 *μ*g/mL were vancomycin-intermediate* Staphylococcus aureus*. Further we had found that 38.6% strains were vancomycin-intermediate* Staphylococcus aureus*. All phenotypically identified methicillin resistant* Staphylococcus aureus* (MRSA) strains possessed* mec*A gene. An extracellular thermostable endonuclease (TNase) which cuts both DNA and RNA was produced by* Staphylococcus aureus* characterized by its gene* nuc* specific for* Staphylococcus aureus* [21]. Eighty-four % strains were found to harbour* nuc* gene. PCR analysis also revealed 40 strains to carry* hlb* gene responsible for beta hemolysis.

## 4. Discussion

In the present study a total of one hundred (60.60%) isolates of clinical bacterial samples from Male Surgical Wards, Orthopedic Ward, and Burn and Wound Section of Midnapore Medical College and Hospital, Midnapore, West Bengal, India, were confirmed to be* Staphylococcus aureus*. Vijayalakshmi [30] reported that a surgical site infection by* Staphylococcus aureus* has found 43.2% in Hyderabad, India. Gram staining, catalase, coagulase, and thermonuclease were important phenotypic identifying markers of* Staphylococcus aureus* [1]. In this study we found that 92% isolates were coagulase positive but the rest of the strains were coagulase-negative, though they were found to be* Staphylococcus aureus* confirmed by PCR analysis using 16S rRNA primers which is specific for this species. This finding is correlated with the findings of Korman [31]. Matthews et al. and Rukumani et al. [32, 33] also found atypical coagulase-negative* Staphylococcus aureus* from food and clinical specimen, respectively. Sanford et al. [34] reported that most clinical strains of* Staphylococcus aureus* associated with pneumonia produced thermostable DNase (88%) and protease (94%). Present study showed that 84% and 81% of the clinical strains produce thermostable DNase and protease, respectively.* Staphylococcus aureus* resistant to methicillin are life threatening bacteria in hospital settings [5]. Studies show that the majority of MRSA strains are associated with hospital-acquired colonization and infections [35]. All* Staphylococcus aureus* strains isolated in this study were multidrug resistant. PCR amplification of* mec*A gene was positive in the 70% of isolates. Khan et al. [36] reported that* mec*A harbouring multidrug resistant* Staphylococcus aureus* strains were found in hospital personnel in a premier hospital in North India. Prevalence rate of MRSA was 44% in a Tertiary Care Hospital, AIIMS, New Delhi [35]. In the present study we have found that the prevalence rate of MRSA is 70%. This finding is supported by Boucher and Corey [5]. They reported that the frequency of methicillin-resistant* Staphylococcus aureus* (MRSA) infections increases continuously in hospital-associated infection. Increased prevalence of infection due to MRSA led to the use of glycopeptides for therapeutic purpose [10]. We found that 54.3% of the methicillin-resistant* Staphylococcus aureus* isolates were also resistant to vancomycin. Vancomycin resistance was also found from intensive care units of tertiary care hospitals in Hyderabad among MRSA isolates [37]. Yaseen et al. [38] reported high prevalence of VRSA among multidrug-resistance MRSA from Al-Jumhuory Teaching Hospital Patients in Mosul, Iraq, and that is similar to our results. Tiwari and Sen [39] also reported the emergence of vancomycin resistant* Staphylococcus aureus* (VRSA) from the northern part of India. The* nuc* gene is used for identification for* Staphylococcus aureus* strains [21]. Among the 100 clinical samples, those that were identified as* Staphylococcus aureus* 84% were found to be* nuc* positive. Sahebnasagh et al. [40] found 80.2% isolates to be* nuc* positive in hospitals of Tehran, Iran. In our study the percentage of* hlb* gene in* Staphylococcus aureus* isolates was 40. This study can be compared with the study performed by Rusenova et al. [41] who showed that thirty-one MRSA isolates (42.5%) were positive for beta toxin. Our data though could not cover the entire population; however, at least in the premise of this hospital, it can be said that MRSA and VRSA are prevalent in the hospital environment. Hereby, it is suggested that there should be periodic review of hospital-associated infections including antimicrobial sensitivity testing. It would be helpful in making antibiotic policy for infection control and reducing the incidence of multidrug resistant MRSA and VRSA.

## 5. Conclusion

The current study depicts the high level of multidrug resistant* Staphylococcus aureus* infections in Surgical Ward, Orthopedic Ward, and Burn and Wound Section of a hospital environment. The emergence of multidrug resistant forms of pathogenic* Staphylococcus aureus* is a worldwide problem in clinical medicine. The prevalence of MRSA isolates is increasing in hospital settings and these isolates are more resistant to vancomycin than previously isolated MRSA. A rapid, conventional identification method, based on phenotypic and genotypic characters, is suitable in clinical laboratory procedures.

## Figures and Tables

**Table 1:** Primer sequences of 16S rRNA, *mec*A, *nuc,* and *hlb* genes of *Staphylococcus aureus*.

Primer	Direction	Sequences (5′-3′)	Amplicon sizes (bp)	Reference
16S rRNA	F	GTA GGT GGC AAG CGT TAT CC	228	[19]
	R	CGCACATCAGCGTCAG		

*mec*A	F	GTG AAG ATA TAC CAA GTG ATT	147	[20]
	R	ATG CGC TAT AGA TTG AAA GGA T		

*nuc*	F	GCGATTGATGGTGATACGGTT	270	[21]
	R	AGCCAAGCCTTGACGAACTAAAGC		

*hlb*	F	GTGCACTTACTGACAATAGTGC	309	[22]
	R	GTTGATGAGTAGCTACCTTCAGT		

**Table 2:** Phenotypic characteristics for identification of *Staphylococcus aureus*.

Source of the isolates	Number of examined samples	Number of *S. aureus* isolates	Colony pigment			Gram stain Number^*∗*^ (%)	Catalase activity Number^*∗*^ (%)	Coagulase activity Number^*∗*^ (%)	Tnase activity Number^*∗*^ (%)
			White	Creamy	Yellow				
			Number^*∗*^ (%)	Number^*∗*^ (%)	Number^*∗*^ (%)				
Male Surgical Ward	80	45	5 (11)	15 (33)	25 (55)	45 (100)	45 (100)	42 (93)	37 (83)
Orthopedic Ward	70	50	18 (36)	12 (24)	20 (40)	50 (100)	50 (100)	45 (90)	42 (83)
Burn and Wound Section	15	5	0 (0)	2 (40)	3 (60)	5 (100)	5 (100)	5 (100)	5 (100)
Total	165	100	23 (23)	29 (29)	48 (48)	100 (100)	100 (100)	92 (92)	84 (84)

^*∗*^Positive number; percentage is presented in parenthesis.

**Table 3:** Important biochemical markers of *Staphylococcus aureus* isolates.

Source of the isolates	Mannitol fermentation with high salt concentration	Gelatinase activity Number^*∗*^ (%)	Urease activity Number^*∗*^ (%)	Protease activity Number^*∗*^ (%)	Lipase production Number^*∗*^ (%)	Esculin hydrolysis Number^*∗*^ (%)
	Number^*∗*^ (%)					
Male Surgical Ward	45 (100)	33 (73)	34 (76)	36 (80)	22 (49)	5 (11)
Orthopedic Ward	50 (100)	40 (80)	35 (70)	41 (82)	25 (50)	5 (10)
Burn and Wound Section	5 (100)	5 (100)	4 (80)	4 (80)	4 (80)	0 (0)
Total	100 (100)	78 (78)	73 (73)	81 (81)	51 (51)	10 (10)

^*∗*^Positive number; percentage is presented in parenthesis.

**Table 4:** Hemolytic activity of clinical isolates of *Staphylococcus aureus*.

Source of the isolates	Number of examined samples	Alpha	Beta	Gamma
		Number^*∗*^ (%)	Number^*∗*^ (%)	Number^*∗*^ (%)
Male Surgical Ward	45	25 (56)	15 (33)	5 (11)
Orthopedic Ward	50	18 (36)	20 (40)	12 (24)
Burn and Wound Section	5	0 (0)	5 (100)	0 (0)
Total	100	43 (43)	40 (40)	17 (17)

^*∗*^Positive number; percentage is presented in parenthesis.

**Table 5:** Susceptibility and resistance pattern of *Staphylococcus aureus *to 8 antimicrobial agents.

Antibiotics	% resistant	% sensitive
Ampicillin (30 mcg)	96	4
Chloramphenicol (30 mcg)	57	53
Streptomycin (10 mcg)	75	25
Erythromycin (10 mcg)	81	19
Kanamycin (30 mcg)	74	26
Nalidixic acid (30 mcg)	89	11
Novobiocin (30 mcg)	75	25
Cefoxitin (30 mcg)	70	30

**Table 6:** Minimum inhibitory concentration of vancomycin of *Staphylococcus aureus *clinical isolates.

Vancomycin MIC (*μ*g/mL)	MRSA (*N* = 70)			MSSA (*N* = 30)		
	VSSA Number^*∗*^ (%)	VISA Number^*∗*^ (%)	VRSA Number^*∗*^ (%)	VSSA Number^*∗*^ (%)	VISA Number^*∗*^ (%)	VRSA Number^*∗*^ (%)
0.25	0 (0)	0 (0)	0 (0)	0 (0)	0 (0)	0 (0)
0.5	3 (4.28)	0 (0)	0 (0)	18 (60)	0 (0)	0 (0)
1	2 (2.85)	0 (0)	0 (0)	12 (40)	0 (0)	0 (0)
2	0 (0)	0 (0)	0 (0)	0 (0)	0 (0)	0 (0)
4	0 (0)	10 (14.3)	0 (0)	0 (0)	0 (0)	0 (0)
8	0 (0)	17 (24.3)	0 (0)	0 (0)	0 (0)	0 (0)
16	0 (0)	0 (0)	36 (51.4)	0 (0)	0 (0)	0 (0)
32	0 (0)	0 (0)	2 (2.9)	0 (0)	0 (0)	0 (0)
Total	5 (7.1)	27 (38.6)	38 (54.3)	30 (100)	0 (0)	0 (0)

^*∗*^Positive number; percentage is presented in parenthesis; VSSA: vancomycin-susceptible *Staphylococcus aureus*; VISA: vancomycin-intermediate *Staphylococcus aureus*; VRSA: vancomycin resistant *Staphylococcus aureus*; MRSA: methicillin-resistant *Staphylococcus aureus*; MSSA: methicillin-susceptible *Staphylococcus aureus*.
